# Early heart rate variability evaluation enables to predict ICU patients’ outcome

**DOI:** 10.1038/s41598-022-06301-9

**Published:** 2022-02-15

**Authors:** Laetitia Bodenes, Quang-Thang N’Guyen, Raphaël Le Mao, Nicolas Ferrière, Victoire Pateau, François Lellouche, Erwan L’Her

**Affiliations:** 1 Service de Médecine Intensive et Réanimation, Medical Intensive Care, CHRU de Brest-La Cavale Blanche, 29609 Brest Cedex, France; 2 Oxynov Inc, Technopole Brest Iroise, 135 rue Claude Chappe, 29280 Plouzané, France; 3 LATIM INSERM UMR 1101, FHU Techsan, Université de Bretagne Occidentale, Brest, France; 4 EA 3878, Département de Médecine Interne, Vasculaire et Pneumologie, Université de Bretagne Occidentale, CHRU de Brest-La Cavale Blanche, Brest, France; 5 Centre de Recherche de l’Institut, Universitaire de Cardiologie et de Pneumologie de Québec, Québec, Canada

**Keywords:** Epidemiology, Preclinical research, Data mining, Predictive medicine

## Abstract

Heart rate variability (HRV) is a mean to evaluate cardiac effects of autonomic nervous system activity, and a relation between HRV and outcome has been proposed in various types of patients. We attempted to evaluate the best determinants of such variation in survival prediction using a physiological data-warehousing program. Plethysmogram tracings (PPG) were recorded at 75 Hz from the standard monitoring system, for a 2 h period, during the 24 h following ICU admission. Physiological data recording was associated with metadata collection. HRV was derived from PPG in either the temporal and non-linear domains. 540 consecutive patients were recorded. A lower LF/HF, SD2/SD1 ratios and Shannon entropy values on admission were associated with a higher ICU mortality. SpO2/FiO2 ratio and HRV parameters (LF/HF and Shannon entropy) were independent correlated with mortality in the multivariate analysis. Machine-learning using neural network (kNN) enabled to determine a simple decision tree combining the three best determinants (SDNN, Shannon Entropy, SD2/SD1 ratio) of a composite outcome index. HRV measured on admission enables to predict outcome in the ICU or at Day-28, independently of the admission diagnosis, treatment and mechanical ventilation requirement.

**Trial registration:** ClinicalTrials.gov identifier NCT02893462.

## Introduction

Cardiovascular function homeostasis is mostly regulated by the autonomic nervous system (ANS) and its sympathetic and parasympathetic branches, thus allowing the organism to maintain its balance when experiencing stress and/or any form of aggression^1^. These regulatory mechanisms are responsible for continuous variation, from second to seconds, in the cardiac function (heart rate and cardiac output) of healthy subjects, even at rest^2–4^. Several studies since the 1960s have suggested a link between the ANS and cardiovascular mortality^5–8^. According to European Society of Cardiology and the North American Society of Rythmology and Electrophysiology guidelines, the fall of HRV seems to indicate an over-cardiovascular mortality^3^. Other studies have also highlighted that HRV variations were correlated to a worse patients’ outcomes in various conditions^9–12^, and the fact that informations about several pathological conditions may be hidden in HRV variations such as during myocardial infarction, pediatric sepsis, fetal distress. However, most of these studies were retrospective and included a small sample size.

If a diagnostic based on HRV seems interesting while it is inexpensive and noninvasive, it is not simple to implement in clinical routine while it requires the access to the raw electrocardiogram (ECG) or plethysmogram (PPG) signals^13,14^, which is not simple within general wards. *A contrario*, patients admitted to ICUs are permanently monitored and multiple physiological variables such as ECG, pulse oximetry, temperature and respiration are continuously recorded, all these parameters being controlled by the ANS.

Based on a prospective data-warehousing project using data-mining and artificial intelligence promoted by Brest University Hospital and LATIM INSERM UMR 1101, the present study was conducted to determine the best determinants of ANS variations in terms of ICU patients’ outcome prediction.

## Results

548 consecutive patients were included in the database, and HRV measurements were available in 540 cases. Comparison between physiological characteristics according to ICU and Day-28 mortality are presented in Table 1. Patients deemed to survive in the ICU environment and at Day-28 were significantly younger and had lower severity scores. Hypoxemia, mechanical ventilation requirement and hemodynamic failure were significantly associated with higher mortality in the ICU and at Day-28.

**Table 1:** Comparison between physiological characteristics, according to ICU and Day-28 mortality.

	All (n = 540)	ICU survival (n = 418)	ICU death (n = 122)	Day-28 survival (n = 418)	Day-28 death (n = 122)
**Physiological characteristics**					
Age (years)	64 [62–65]	**63 [53–71]**	67 [58–75]*	**62 [51–71]**	67 [59–75]**
Sex ratio, n female (%)	185 (34.3%)	141 (34.1)	43 (35.2)	132 (31.6)	43 (28.9)
Actual body weight (kg)	74 [72–75]	74 [64–88]	72 [64–88]	75 [64–88]	72 [64–87]
IMC (kg/m^2^)	25.8 [25.1–26.3]	25.8 [22.4–30.0]	26.0 [22.8–30.5]	25.8 [22.5–30.0]	26.0 [22.8–30.1]
IGS II	51 [48–53]	**47 [33–62]**	61 [46–76]***	**46 [33–61]**	62 [46–76]***
Body temperature (°C)	37.0 [36.7–37.5]	**37.2 [34.4–38.1]**	36.6 [34.4–38.1]*	**37.3 [36.2–38.2]**	36.6 [35.1–38.1]**
Glasgow Coma Scale	14 [13–14]	**14 [8–15] **	7 [3–14]***	**14 [9–15]**	7 [3–15]***
Heart rate (b/min)	91 [89 – 93]	90 [77–106]	91 [74–105]	90 [77–106]	91 [74–105]
Mean arterial pressure (mm Hg)	82 [80 – 83]	**83 [73–94]**	75 [68–90]**	**83 [73–94]**	77 [68–92]**
Respiratory rate (c/min)	22 [21–23]	20 [18–25]	22 [18–27]	**20 [17–25] **	22 [18–28]*
SpO_2_ (%)	95 [94 – 95]	95 [93–97]	95 [92–97]	95 [93–97]	95 [92–97]
SpO_2_/FIO_2_	372 [347–384]	**387 [287–457]**	300 [209–430]***	**387 [283–457]**	300 [225–442]***
PaO_2_/FIO_2_	228 [200–237]	232 [174–328]	226 [143–305]	230 [171–324]	226 [143–300]
**Pathological characteristics**					
Actual ICU admission cause					
Respiratory failure, n (%)	212 (39.3)	162 (39.2)	45 (36.9)	152 (36.4)	52 (42.6)
Cardiovascular failure, n (%)	172 (31.9)	**118 (28.6)**	54 (44.3)**	**107 (25.6)**	61 (50.0)**
Neurological failure, n (%)	120 (22.2)	**99 (24.0)**	19 (15.6)*	**96 (23.0)**	22 (18.0)*
Metabolic and other, n (%)	38 (7.0)	34 (8.2)	4 (3.3)	33 (7.9)	4 (3.3)
Associated pathological status					
Unstable hemodynamics, n (%)	77 (14.3)	**45 (10.9)**	31 (25.4)**	**41 (9.8)**	31 (25.4)**
Vasopressive agents, n (%)	150 (27.8)	**91 (22.0)**	57 (46.7)***	**85 (20.3)**	61 (50.0)***
Anti-hypertensive agents, n (%)	31 (5.7)	26 (6.3)	5 (4.2)	26 (6.2)	5 (4.1)
Fluid expansion during the 24 h (mL)	0 [0–0]	0 [0–500]	0 [0–500]	0 [0–500]	0 [0–500]
Atrial fibrillation or sustained arrhythmia, n (%)	77 (14.3)	57 (13.8)	17 (13.9)	54 (12.9)	18 (14.8)
Dialysis, n (%)	217 (40.2)	**156 (39.0)**	61 (50.0)*	159 (39.0)	58 (47.5)
ECMO, n (%)	6 (1.1)	4 (1.0)	2 (1.6)	4 (1.0)	2 (1.6)
Immune-depression, n (%)	45 (8.3)	29 (5.8)	15 (12.3)	29 (5.8)	15 (12.3)
Cardiac history, n (%)	280 (51.9)	209 (50.6)	68 (55.7)	192 (45.9)	80 (65.6)
Respiratory history, n (%)	196 (36.3)	149 (36.2)	43 (35.2)	141 (33.4)	48 (39.3)
Respiratory support					
Spontaneous ventilation, n (%)	212 (39.3)	**192 (46.5)**	18 (14.8)***	**178 (42.6)**	28 (23.0)***
O_2_ flow under spontaneous ventilation (L/min)	0 [0–0]			0 [0–2]	3 [0.11]
High nasal flow oxygen, n (%)	24 (4.4)	17 (4.0)	7 (5.7)	15 (3.6)	8 (6.6)
Gas flow under HNFO (L/min)	50 [50–60]				
Non-invasive ventilation, n (%)	20 (3.7)	17 (4.0)	3 (2.6)	17 (4.1)	3 (2.5)
Invasive mechanical ventilation, n (%)	286 (53.0)	**187 (45.3)**	94 (77.0)***	**178 (42.6)**	100 (82.0)***
Sedation	57 (10.6)	**34 (8.2)**	20 (16.4)*	**30 (7.2)**	20 (16.4)*
Paralyzing agents	21 (3.9)	**10 (2.4)**	10 (8.2)*	**10 (2.4)**	10 (8.2)*
Patient/ventilator asynchrony, n (%)	78 (14.4)	**61 (29.2)**	17 (17.9)*	54 (12.9)	24 (19.6)
Active humidification, n (%)	130 (24.1)	91 (49.5)	37 (56.5)	89 (21.3))	39 (32.0)
FIO_2_ (%)	30 [30–30]	**30 [21–40]**	35 [25–50]**	**30 [21–40] **	31 [21–45]*
Ventilatory rate (c/min)	19 [18–20]	**18 [15–21] **	20 [16–24]*	19 [15–22]	20 [15–24]
Tidal Volume (mL/kg IBW)	7.2 [7.1–7.3]	7.3 [6.7–7.8]	7.0 [6.5–7.6]	7.2 [6.7–7.8]	7.2 [6.5–7.7]
PEEP (cmH_2_O)	5 [5–5]	5 [4–5]	5 [4–5]	5 [4–5]	5 [4–5]
**Outcome parameters**					
Mechanical ventilation duration	3 [2–3]	**2 [0–7]**	6 [3–12]***	**2 [0–7]**	5 [1–10]***
Respiratory support duration	3 [3–4]	**3 [0–7]**	6 [3–13]***	**3 [0–8]**	5 [2–10]**
ICU LOS (days)	7 [6–7]	**6 [4–12]**	9 [4–18]*	6 [4–14]	8 [4–14]
Hospital LOS (days)	19 [17–21]	**21 [10–44]**	12 [5–25]***	**22 [10–46]**	12 [5–22]***

Results are provided as Median [95% CI for the Median], or Number (%), unless specified otherwise.

For the head of columns, the number of patients in between parenthesis represent the number of patients for whom the parameter was available for monitoring.

Unstable hemodynamics was defined as the need for > 1 L fluid expansion and/or vasopressive agents introduction/posology modification within the previous 24-h; Patient/Ventilator Asynchrony was detected at the patient’s bedside during data monitoring and considered if the Asynchrony Index was higher than 10%; Respiratory support was defined as either the use of invasive mechanical ventilation, noninvasive ventilation and high nasal flow oxygen therapy.

*BMI* body mass index, *Day 28* mortality was assessed at day 28, *ECMO* extracorporeal membrane oxygenation, *FIO*_*2*_ inspired oxygen fraction, *LOS* length of stay, *SAPS II* simplified acute physiological score II, *SpO*_*2*_ pulse oximetry, *ICU* intensive care unit.

Significant values are in bold.

HRV comparison in between death and survival within the ICU and at Day-28 are provided within Table 2. Death in the ICU and at Day-28 was associated with lower LF/HF (ratio Low Frequency/High Frequency), SD2/SD1 and Shannon entropy values on admission.

**Table 2:** Comparison between HRV values measured on admission, according to ICU and Day-28 mortality.

	All (n = 540)	Survival ICU (n = 418)	Death ICU (n = 122)	Day-28 survival (n = 418)	Day-28 death (n = 122)
**Temporal domain**					
SDNN (ms)	30.2 [25.8–34.8]	30.4 [12.6–89.4]	27.4 [9.3–110.3]	30.7 [12.3–93.4]	28.4 [10.7–106.6.3]
RMSSD (ms)	33.8 [28.0–40.5]	32.8 [15.3–100.7]	31.3 [13.1–128.4]	32.7 [14.8–101.2]	35.7 [14.1–122.8]
pNN50 (%)	6.9 [5.1–9.8]	38.8 [5.5–179.8]	34.9 [2.7–201.1]	38.8 [5.3–187.3]	39.0 [4.7–205.8]
Triangular Index	5.3 [4.8–6.0]	5.4 [3.1–10.3]	4.6 [2.7–13.5]	5.5 [3.1–11.6]	4.8 [2.8–11.8]
**Frequency domain**					
VLF (0.0–0.04 Hz) (ms^2^)	55.9 [32.5–81.3]	63.3 [6.3–450.2]	24.0 [2.7–471.2]	65.2 [6.0–468.7]	32.8 [3.7–463.0]
LF (0.04–0.15 Hz) (ms^2^)	390.5 [203.3–616.0]	443.5 [36.2–4543.0]	162.3 [13.6–6134.7]	452.5 [35.1–5260.8]	268.3 [25.0–6443.7]
HF (0.15–0.4 Hz) (ms^2^)	301.0 [227.5–497.9]	303.0 [46.3–4878.5]	252.0 [25.5–7385.9]	301.0 [43.9–5509.3]	333.5 [36.7–7388.3]
LF/HF	0.97 [0.56–1.57]	**1.02 [0.62–1.61]**	0.77 [0.37–1.34]**	**1.02 [0.60–1.61]**	0.86 [0.42–1.33]*
**Non linear domain**					
*Entropy*					
Approximate entropy	1.19 [1.02–1.39]	1.20 [1.03–1.39]	1.14 [0.99–1.37]	1.21 [1.03–1.39]	1.14 [0.97–1.38]
Sample entropy	1.52 [1.13–1.75]	1.55 [1.14–1.77]	1.35 [1.11–1.70]	1.54 [1.15–1.77]	1.37 [1.09–1.71]
Shannon entropy	3.06 [2.76–3.50]	3.09 [2.79–3.52]*	**2.97 [2.65–3.33]**	**3.09 [2.77–3.51]**	3.00 [2.68–3.45]*
*Poincaré analysis*					
SD1 (ms)	23.3 [19.7–28.2]	23.2 [10.9–71.3]	22.2 [9.3–90.9]	23.2 [10.6–74.0]	25.3 [10.0–86.9]
SD2 (ms)	33.2 [28.2–40.0]	33.3 [13.3–101.3]	28.5 [9.7–123.1]	33.4 [12.9–109.4]	31.4 [10.9–121.5]
SD2/SD1	1.31 [1.04–1.57]	**1.34 [1.08–1.60]**	1.18 [0.94–1.45]**	**1.33 [1.06 – 1.60]**	1.26 [0.97–1.52]*

*HF* high frequency, *LF* low frequency, *LF/HF* ratio of low frequency to high frequency, *NN* normal interbeat, *pNNx* percentage of consecutive NNs that vary by more than “x” ms, *RMSSD* root mean square of successive differences, *SD1* standard deviation 1, *SD2* standard deviation 2, *SD2/SD1* ratio SD2/SD1, *SDNN* standard deviation of NN, *VLF* very low frequency.

Significant values are in bold.

*P ≤ 0.05; **P ≤ 0.005; ***P ≤ 0.0001.

Multivariate analysis enabled to detect three independent ICU survival predictor: SpO_2_/FiO_2_ (OR = 2.73; P = 0.007) and two HRV parameters (LF/HF [OR = 2.14; P < 0.001] and Shannon entropy [OR = 1.82; P < 0.005]). While excluding extreme Glasgow and SpO2 values (GCS < 7 and SpO_2_ > 96%), Shannon entropy and SpO_2_/FIO_2_ remained highly associated with ICU survival (OR = 3.79 and 3.35 respectively; P < 0.005 for both). Only SpO_2_/FIO_2_ was considered as an independent Day-28 survival predictor (OR = 3.96; P < 0.0001).

**Figure 1:**
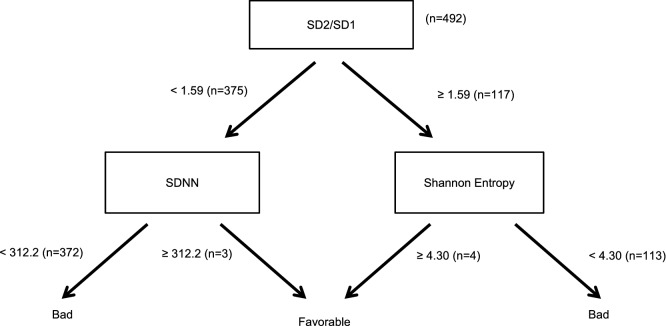
Decision tree for outcome evaluation following admission in the ICU. *NN* normal interbeat, *SD1* standard deviation 1, *SD2* standard deviation 2, *SD2/SD1* ratio SD2/SD1, *SDNN* standard deviation of NN, *Bad* a bad outcome was defined as either death and/or any form of respiratory assistance and/or a median ICU stay higher than the median, *Favorable* a favorable outcome was defined as the occurrence of none of the previous adverse events.

Machine-learning was performed on a bundle of 492 individual data to determine the best predictors for the ICU composite prognosis index; results are provided as a simple decision tree (Fig. 1), combining 3 temporal and non-linear HRV methods (SDNN (Standard deviation of normal interbeat), Shannon Entropy, SD2/SD1 ratio). Discrimination is performed at the first step for most patients, the second step being used to eliminate false signals. Positive and negative predictive values of this decision tree are 99.1 and 83.3% respectively, with a sensitivity Se = 99.8% and a specificity Sp = 5.2%.

## Discussion

In this prospective study using automatic data features extraction from a data warehousing project and artificial intelligence by neural networking, a simple physiological parameter (SpO_2_/FIO_2_) and two HRV parameters (LF/HF and Shannon Entropy) measured on admission were found accurate to predict patient’s outcome. AI and machine-learning enabled to develop an ICU composite outcome predictor, using HRV parameters solely.

HRV analysis is a well-known technique to analyze the autonomous nervous system activity and it is believed to correspond to the balance between the sympathic and parasympathic influences on the intrinsic rhythm of the sinoatrial node. Its first description was performed in the early 1960’s in neonatology^15^. According to European Society of Cardiology (ESC) and North American Society of Pacing and Electrophysiology (NASPE), HRV decrease appears to be a reliable risk marker for cardiovascular mortality^3^. This technique also demonstrated its effectiveness as a marker of mortality and multi-visceral failure syndrome^9,10,16,17^.

Patients admitted to ICUs are permanently monitored for temperature, oxygen saturation, respiration, urinary output and ECG. These parameters are most of the time only monitored while setting alarm ranges, in order to trigger the corresponding alarm for any deviation from the normal range. However, many of these variables are controlled by the ANS, which can be affected as already discussed by several pathological processes and/or drugs. The overall accuracy of HRV to predict outcome within the ICU may simplify workflow and enable prompt response to any clinical status change, while all these parameters can be automatically extracted from raw physiological signals that are already routinely monitored.

In the 1990’s, HRV analysis that was only performed using time-based methods (RR interval analysis), encountered a sudden upsurge with the appraisal of spectral and non-linear analysis, especially in the Anesthesiology and the ICU fields. In 1994, Hogue et al. demonstrated in an observational study the breach in pace regulation by SNA during heart surgery post-operative phase, illustrated by a HRV decrease^18^. More recently, Lakusik et al. included 206 patients following surgical coronary artery bypass, found an increased mortality among patients with decreased HRV^19^. In the ICU, the HRV reduction has already been correlated to an increased mortality on a 746 patients’ sample, supporting the hypothesis that HRV is a clinically relevant variable in such settings^20^. More recent ICU studies have been mainly focused on HRV measurements in septic patients^11,21–23^ and depicted a link between HRV alteration in the early stage of sepsis and the pejorative evolution of the patients. In a prospective study performed on 96 septic patients under vasopressor drugs, HRV monitoring during amines titration enabled to decrease the total amount of drugs needed for hemodynamic balance^24^. Multiple studies performed on trauma patients also confirmed this correlation between low HRV and short-term poor prognosis^25–27^. HRV measurement was also demonstrated to be effective during ventilatory weaning^28,29^, acute stroke^30^, delirium prediction^31,32^, and pain management^33,34^. In 2017 in a multicenter randomized trial of 70 ICU patients, Sunil et al. studied the possibility of a customized algorithm based on HRV measure to manage patient sedation^35^. They modified HRV measurement according to sedation level, thus establishing a new framework for patient sedation management in order to limit the harmful effect of a deep sedation.

When considering the available variables for HRV analysis, several characteristics may be extracted, either in the temporal, frequency or fractal domain. While simpler to compute than other parameters, the temporal domain characteristics of the cardiac rhythm do not necessarily indicate whether a change in HRV had been caused by the sympathetic or the para-sympathetic system. Within our database, none of these temporal domain parameters were considered as associated to outcome. Frequency domain analyses seems to be better equipped than temporal domain to discriminate the individual contribution of the sympathetic/parasympathetic systems, as they manifest themselves in two non-overlapping frequency bands (LF, HF), and considering these arguments, the LF/HF ratio has gained a wide acceptance as a tool to assess cardiovascular autonomic regulation. However, the LF peak of the heart rate power spectrum which is often assumed to have a dominant sympathetic component (the HF peak reflecting the cardiac parasympathetic nerve activity), is complex to evaluate as it may at least be modified by various stimulus and medications^36–38^. It is also established that increases in respiratory frequency reduce the amplitude of heart rate oscillations^39^ while increases in tidal^39,40^ or static lung volume^41^ provoke increases in the R–R interval variability; the influence of mechanical ventilation within the ICU may therefore have a major influence on HRV variation in such environment. Considering such frequently under-appreciated limitations, HRV frequency domain evaluation may rather reflect a complex and not easily discernible mix of sympathetic, parasympathetic, but also the impact of other unidentified factors^42^. Despite all these limitation, lower LF/HF ratios on admission were significantly associated to a lesser outcome within our ICU population. The VLF peak is a third part of the frequency spectrum that has been less evaluated, but that seems to be associated to prognosis in various pathologies, such as during CHF^43^. Considering that the R-R intervals are non-linear, it is also needed to measure it using non-linear methods. In other ways, nonlinear methods are used to observe regularity, predictability, and complexity in the time series while considering the impact of several interventions and the influence pathological processes. The concept of complexity is different from variability,but no consensus definition of what complexity includes do exist. It is often used to characterize the behavior of a non-linear system that contains many parts that interact with other in highly different ways; two signals can have the same degree of variability (i.e., same variance and coefficient of variation), but different complexity properties. Complexity may be approached by the means of entropy measurements or by the Poincaré plot analysis. The Poincaré analysis recognizes the hidden correlation patterns of a time series signal^44^, and it consists in a visual representation and a quantitative measure of the temporal dynamics of the R-R interval. Unlike frequency-domain measurements, Poincaré plot analysis is insensitive to changes in trends in the R–R intervals^45^. Entropy is another approach to measure complexity that is derived from the information theory. While considering the heterogeneity of pathological processes for patients attending the ICU, combined the methodological limitations of one parameter evaluation solely as described earlier, we do emphasize the importance of a model that combines the evaluation of various components of the HRV, associated with individual clinical status evaluation. No single measure is sufficient to capture the properties of the most complex signals; instead, an ensemble of measures is required to probe signals of interest for different attributes. The neural networking derivation of our predictive model is in line with such discussion, while two over three parameters that were selected within the model are derived from the non-linear domain and could thus illustrate the complexity of the pathological processes and evaluate the impact of therapeutic interventions and pathological processes.

Critically ill patients’ care and decision-making is complex, involving interpretation of many variables and comparative evaluation of various therapy options. Many critical care indices are not captured automatically, and relevant data are thus difficult to evaluate, especially while considering human factors like vigilance in a constraint environment, varying expertise of the attending physicians, and the constant need to deal with a high-level of uncertainty under time-constraints. The lack of standardization for the different methods of HRV measurements in the critical care setting may also require specific attention, thus requiring adequate parameters selections that would consider either technical consideration, but also patients’ complexity and the potential impact of several pathological processes and therapeutic interventions as mentioned earlier.

Knowledge-based decisions using AI is a new area within the ICU that may help in solving several decision problems in a difficult environment^46^. It could assist the physician in integrating and interpreting simultaneously various data sources, thus potentially enabling timelier and more targeted interventions. Considering our results, neural networking was able to develop a prediction model using HRV parameters solely that enabled to predict outcome within the ICU, while considering various parameters such as the survival, the need for respiratory assistance and a potentially longer length-of-stay. However, when applying the decision tree to real data, we were able to depict rather high positive and negative predictive values, a very high sensibility, but with a rather low specificity. Beside a prospective validation of the decision tree, such low specificity will certainly require to consider the tool more as an alarm, than as a clinical decision tool.

Heart Rate Variability analysis may be considered as an additional tool, in line with our usual critical patient monitoring. Its low cost and high efficiency, demonstrated for several years in the literature, through different fields of medicine, make its use a serious avenue and seems possible in terms of patient triage eligible for critical care, for emergency services or as an early warning sign in a resuscitation patient situation. In addition, HRV is a simple parameter immediately available in comparison to a multimodal grid that is long to fill when not automated that is SAPSII calculation, which is a static tool unlike the HRV which is a dynamic tool. As stated within a previous study from our team^47^, the use of PPG is ubiquitous, not only in ICUs but also in most general wards or step-down facilities, while continuous ECG monitoring is limited to ICUs. If continuous ECG monitoring may of course enable ICU HRV analysis by its integration within the monitoring units, our current approach of HRV monitoring based on the PPG signal may thus facilitate monitoring and early-warning evaluation in various clinical settings, through the integration of the algorithm models within more simple biomedical devices, with less sensors attached to the patient.

Several limits of the study may be discussed. First, the current gold standard for HRV measurement is the ECG, whose principle is based on obtaining a potential difference between the electrodes. Nevertheless, standard ECG monitoring is not able to gather electrical potentials created by the activation of atrial and ventricular myocardium. The sinus node, the atrio-ventricular node, the Hiss bundle and its branches remain silent on a standard plot, because their potentials amplitudes are too weak, except while using high amplification methods. On the other side, respiratory PPG has the undeniable advantage of its robustness and simplicity of use. In addition to its role in oximetry monitoring, its use is growing in the field of noninvasive hemodynamic monitoring. We do emphasize the fact that PPG signal may be affected by several factors such as age, arterial stiffness as examples, and may thus be less precise and accurate, at least from a physiological point of view, than HRV evaluation based on ECG. Nevertheless, our results based of PPG signals, depicting statistical correlation of such evaluation with patients’ outcome do enable us to consider the validity of our approach. Second, the monocentric and retrospective analysis design of the study may limit the overall application of these results. However, while the analysis was performed on a retrospective basis, data were collected prospectively, using a non-probabilistic approach. Combined with the number of included patients, one may consider that these biases may be limited. Third, we may also consider that the results may be distorted due to many confounding factors such as the mode of admission, mechanical ventilation requirement, vasopressor drug’s needs, or patients’ co-morbidity. However, the general characteristics of the patients’ sample depicts general distribution of patients in a standard medical ICU. Moreover, the results that are presented herein are consistent with previous analysis of the literature. Within our results, the SpO_2_/FiO_2_ ratio was depicted as significantly correlated with mortality while survivors did not differ from non-survivors in terms of respiratory failure diagnosis upon admission. Such results may typically be related to the huge complexity of pathological processes within the ICU, and the fact that other pathologies than respiratory diseases may induce SpO_2_/FiO_2_ ratio. Fourth, while we were able to depict a potential interest of HRV measured on admission to predict ICU outcome, the question whether such parameters might enable continuous monitoring and treatments efficacy measurement and/or therapeutic adjustments remains unanswered.

## Methods

### Study design and population

Data analysis was performed on the ReaSTOC database, which is an ongoing prospective physiological and biomedical signal data warehousing project including all consecutive patients admitted to our adult medical ICU (ClinicalTrials.gov identifier NCT02893462). A previous publication has described the design and conduct of the ReaSTOC study^47^. The protocol was approved by local ethics committee (*Comité d’Ethique du CHRU de Brest 2018CE.27)* and written informed consent was waived according to French legislation (Law n° 2012–300 March 5^th^ 2012 also called “Loi Jardé” https://www.legifrance.gouv.fr/eli/jo/2012/3/6). All physiological data were collected by the same research assistant, using a standardized protocol. Clinical metadata were recorded using standard monitoring devices and the computerized medical file (Intellivue MP70® and ICCA® Philips Healthcare, Amsterdam, Netherlands). The only exclusion criteria was the patient and/or relatives refusal to participate.

### Measurement

#### General data recording

Each raw physiological PPG plot was recorded in native resolution (75 Hz) during 2 consecutive hours, within the first 24 h following ICU admission, using a private data network and a dedicated software (Synapse, LTSI INSERM UMR 1099). All measurements were performed with the patients lying in a supine position, with a 30° angulation of the upper part of the body.

Clinical metadata included general and physiological parameters, among which, the history of diabetes and treatment with beta-blockers, which may influence the patient’s heart rate variability. Outcome parameters included length-of-stay and survival, which parameters were evaluated either during the ICU and Hospital (ICU and after discharge to General Wards), or at Day28 (ICU, General Ward, and potentially after hospital discharge).

Length-of-stay in ICU was defined by the number of days spent in ICU.

If the patient was admitted to the ward after being discharged for more than 48 h, we would consider a new hospitalization.

The length of hospitalization was defined by the entire amount of time spent in the hospital.

Hospital mortality was collected on the living or dead status on discharge, regardless of the length-of-stay.

The status at Day 28 was collected on the hospital software and if this information was not specified, the patient’s attending physician was contacted.

We also used a composite prognosis index for outcome evaluation that was determined a priori as: 1-death, 2- and/or the need for any form of respiratory assistance during the ICU stay, 3- and/or a ICU length-of-stay higher than the median duration of 7-days.

Hemodynamic instability was defined as the need for a fluid challenge (≥ 0.5 L crystalloids) and/or introduction or modification of vasopressive drugs within the past 2 h of care.

#### Features extraction

According to previous publication using the same database^47^, HRV measurements were performed on PPG. All tracings were analyzed using Kubios HRV Premium 3.0 (Kuopio, Finland), using standard methods (see Addendum). For each marker, we considered the average value of 3 data samples of at least 3 consecutive minutes, over the two hours of recording.

##### Time based analysis

Time-based analysis was performed using statistical and geometrical methods. Parameters to be analyzed were the R-R intervals, Root Mean Square of Successive Differences (RMSSD), and the triangular index.

##### Frequency-based analysis

Spectral analysis using the discrete Fourier transform identified high frequencies (HF 0.15–0.40 Hz), low frequencies (LF 0.04–0.15 Hz), and very-low frequencies (VLF 0.0033–0.4 Hz) bandwidths^48^, LF/HF ratio was also calculated.

##### Non-linear analysis

Non-linear chaotic evaluation method was performed using the Poincaré plotting, thus providing the standard deviation of instant beat-to-beat time variation (SD1) and the standard deviation of long-term beat-to-beat time variation (SD2)^49^.

Several wavelet entropy parameters were measured such as Approximate Entropy^50^, Sample Entropy, and Shannon Entropy^51^.

### Statistical analysis

Continuous variables were expressed as mean ± SD in case of normal distribution and median (interquartile range (IQR)) otherwise; categorical variables were expressed as numbers and percentages.

Univariate analysis was performed using a non-parametric Kruskall–Wallis test for quantitative and Fisher’s exact test for qualitative datasets. The optimal thresholds of HRV indicators were determined assessing receptor efficiency function (ROC curves). In all cases, we considered a minimal 60% sensitivity and specificity to select the threshold value.

A feature ranking was subsequently performed by discriminant analysis with cross-validation via artificial intelligence (AI) and neural network using kNN (*k-Nearest Neighbors*). The selected features were assessed for their prediction performance with supervised and unsupervised machine learning methods (kNN, subspace discriminant, fine Gaussian SVM, logistic regression, decision trees, K-Means and neural networks).

Positive and Negative Predictive values (PPV, NPV), sensitivity (Se) and Specificity (Sp) of the decision tree were calculated a posteriori when applying the results to the real database.

Statistical significance was defined as a two-tailed *P*-value of < 0.05 for all analyses. Analyses were performed using MedCalc software v17.8 (Ostend, Belgium) for univariate and multivariate analysis, and MATLAB for machine learning and neural-networking (MathWorks, Natick, MA, USA).

### Study approval

This study was conducted following the amended Declaration of Helsinki.

All patients, families or people they trust will be informed of the study and of their possibility of withdrawing consent to use data for research purposes through an information form inserted in the booklet provided at the admission of patients and families in the medical intensive care unit of the CHRU de Brest. The patient's confirmation of participation will be sought from the adult when the latter's condition allows it. The patient's participation will be noted in his medical file.

The ethics Committee of Brest University Hospital approved the study protocol (2018CE.27).

## Conclusion

SpO_2_/FIO_2_ and HRV measured on ICU admission enables to predict outcome either in the ICU and at Day-28, independently of the admission diagnosis, treatment and mechanical ventilation requirements. Simple decision tree including only HRV parameters also enables to predict an ICU outcome, according to a composite index.
